# Sustainable Fisheries Management and the Welfare of Bycaught and Entangled Cetaceans

**DOI:** 10.3389/fvets.2018.00287

**Published:** 2018-11-20

**Authors:** Sarah J. Dolman, Philippa Brakes

**Affiliations:** ^1^Whale and Dolphin Conservation (WDC), Chippenham, United Kingdom; ^2^Centre for Ecology and Conservation, University of Exeter, Penryn, United Kingdom

**Keywords:** cetacean, bycatch, entanglement, welfare, fishing, Europe

## Abstract

The incidental capture of cetaceans and other protected marine wildlife in fishing gear has significant welfare implications. Many thousands of cetaceans are bycaught in fishing gear in European waters and hundreds of thousands die globally. We can expect many more to survive, but suffer from such interactions. As marine policy focuses on “population level” impact assessments and “sustainability” of fishing to preserve fish populations, the impacts to the bycaught individual, and their wider social group, are often largely underestimated, despite the large numbers affected. The wide range of recorded injuries, including abrasions, cuts, bruising, and broken bones, along with the potential for panic associated with forced submersion, indicate that the welfare of bycaught cetaceans is, individually and collectively, very poor. Commercial fishing is the last human activity targeting wildlife (fish) on a grand scale where slaughter includes incidental killing of other large sapient wildlife on such a regular basis. Here, we review the compelling evidence of the short and long term welfare impacts of bycatch, and the progress made toward implementation of measures to understand and solve this significant welfare issue. We argue that policy decisions surrounding fishing do not adequately consider cetacean bycatch, including welfare impacts. Ultimately, there are welfare issues in all bycatch situations and suffering cannot plausibly be reduced without preventing bycatch. The well-documented welfare implications provide a strong argument for zero tolerance of cetacean bycatch and provide a compelling case for immediate action in fisheries where bycatch is taking place. The only way to reduce the suffering of bycaught cetaceans is to decrease, or ideally eliminate, the number of animals caught in fishing gear. Uncertainties around the scale of bycatch should not delay management, even where individual bycatch estimates are considered “sustainable.” Lack of monitoring of sub-lethal impacts on populations may result in flawed impact assessments. We urge that animal welfare considerations should become an integral part of management decision-making in relation to bycatch globally. Enhanced, robust and transparent management systems are urgently required for the range of fisheries within which cetacean bycatch occurs, with the aim to better document and most importantly, work toward eliminating cetacean bycatch altogether.

## Introduction

Bycatch, including entanglement in nets and ropes, is the unintentional capture of non-target species in fishing gear. Each year, hundreds of thousands of whales, dolphins, and porpoises die from incidental capture (1) and many more will survive and suffer from interactions with fishing gear (2). Not only is bycatch a significant conservation issue for a number of species globally, it is a serious and considerable welfare issue.

An International Whaling Commission (IWC) Welfare Workshop held in 2016 (3) emphasized that entanglement in fishing gear is the most significant threat to wild cetacean welfare. Bycatch has wide reaching welfare consequences, affecting quality of life (4–6) for the many whales, dolphins, and porpoises that become injured or suffer the loss of conspecifics. As sentient and highly intelligent beings, cetaceans are considered to be in the highest category on a scale of sensibility to pain and suffering, in the same category as primates and carnivores (7).

Our understanding of the welfare implications of cetacean bycatch has increased, but no quantitative assessment and comparison of the extent of mortality, or the scale of morbidity and welfare implications for bycaught cetaceans between different fisheries exists (2). In this regard, the welfare of bycaught cetaceans is decades behind farm animal welfare and slaughter (8). The animal welfare consequences of the incidental capture of cetaceans and other protected marine wildlife would not be tolerated in terrestrial farming practices (9). Commercial scale fishing is the last human activity targeting wildlife (fish) where slaughter includes incidental take of other large sapient wildlife on such a regular basis and on this scale. Yet, there have been insufficient changes in fisheries management practices and, in general, inadequate effort to reduce the numbers of cetaceans caught in nets generally [for example, (10–12)].

Typically, the focus of research related to cetacean bycatch is that of understanding conservation and population level impacts. Further, assessment of criteria for “eco-labels” focus on the “sustainability” of fish stocks, inadequately cover protected species bycatch, and do not consider welfare at all. Such a narrow view, of both bycatch research and consideration of bycatch in eco-labels, which focus on conservation implications (of the targeted species) and ignore welfare concerns are at odds with the concerns of the general public. The general public assume, inaccurately, that fish certified as “eco-friendly” will also consider and deal with protected species bycatch. This may be part of the explanation why there has been so little action to address bycatch. Public opinion is strong against bycatch^1^ and the public do not accept that cetacean and other protected species bycatch is a tolerable “by-product” of fishing. Whilst an increasing number of fisheries are labeled as “sustainable” in European waters, this assessment focuses on fish stock sustainability. “Sustainable” does not necessarily mean that fisheries can also be considered responsible with regard to bycatch, with variable and often inadequate levels of bycatch assessment, monitoring, and mitigation. The levels of bycatch of cetaceans and other protected species are not well-understood because of poor bycatch management in the majority of fisheries [see, for example, (13–15)] but known to be high in some where monitoring occurs (Birdlife International, in preparation). Hence, buying “sustainable” fish or indeed some fish products labeled “dolphin-friendly” provide no guarantees that incidental bycatch of protected species does not occur alongside the targeted catch^2^. Consumers are concerned with the welfare standards associated with the fish they buy and negative effects for incidentally bycaught species and this is indicated by the growth of such “eco-labels” (16). Perhaps the most recognized example is the Eastern Tropical Pacific tuna-dolphin issue (17), where public outrage and pressure led to better practices and dramatically reduced dolphin bycatch (although problems still remain, identified below). A strong public concern about the welfare of cetaceans and other marine species incidentally caught in fishing gear has been demonstrated. Regardless, a review of Marine Stewardship Council (MSC) fisheries, where MSC is perhaps the best recognized of all existing fisheries certification schemes, has shown that poor bycatch monitoring and reporting hinders assessment of the impact of the majority of reviewed fisheries (28) on bycatch species (Birdlife International, in preparation).

As an indication of the scale of the bycatch problem within European waters, odontocete populations likely to be impacted in some parts of the Northeast Atlantic include harbor porpoise (*Phocoena phocoena*) in static nets (18–23) and in beach seines (24); common dolphin (*Delphinus delphis*) in trawls [(25, 26), and see case study below] and bottlenose dolphin (*Tursiops truncatus*) (27). In the Mediterranean, there is evidence of population level impacts from bycatch on common and striped dolphin (*Stenella coeruleoalba*) (10, 28) and the demographically isolated population of sperm whale (*Physeter microcephalus*) (10) and in the Black Sea in static nets on an endangered sub-species of harbor porpoise, as well as bottlenose and common dolphin (29, 30).

Static fishing pot gear is a significant cause of morbidity and mortality for baleen whales, as well as nets. Entanglement in static fishing gear is the leading cause of detected mortalities of large whales in the Northwest Atlantic (31). Whilst data are limited in European waters, due to a lack of dedicated studies, there are indications that the post-whaling recovery rate of humpback whales (*Megaptera novaeangliae*) in Scottish waters may be hampered by the number of creel entanglements (32). Smaller minke whales (*Balaenoptera acutorostrata*) appear less likely to survive any entanglement than larger species, such as humpback whale (33). The welfare impacts associated with minke whale entanglements are discussed in more detail in the case study below.

Efforts to calculate the “sustainability” of removal through bycatch can be useful to identify those marine mammal populations where bycatch (and other causes of death) are likely to result in population level impacts. For example, in the United States (34–36) and for harbor porpoise in the Baltic Sea, North Sea and Dutch waters (37–39). These studies recognize that there are limitations, biases and caveats to this approach [see for example, (40)]. The impediments to this statistical approach include the considerable uncertainty surrounding population and bycatch data in many parts of the world. Further, the mortality limits focus only on direct mortality and not indirect or sub-lethal effects and their possible population level consequences (39). Such an approach is a useful coarse statutory tool and has a role in identifying situations where bycatch is likely to be causing significant population level effects. As an example, the recent United States (US) Import Rule has been influential in identifying fisheries outside the US that import to the US. These non-domestic imports will be required to meet the bycatch standards of the US's own protected species regulations in coming years (11, 41). Bycatch legislation is, almost without exception, weaker in the rest of the world than in the US, so the US Import Rule is expected to provide an incentive to improve global bycatch measures. However, such an approach provides only part of the solution as the more subtle effects on populations over time require the development of finer-scale management tools and as such, implementation of the US Import Rule and other efforts to assess “sustainability” should be seen as a starting point for ongoing reduction in global bycatch and not as an end-point. Scheidat et al. (39) identify measures (including using the appropriate distribution for the porpoise population, rather than political boundaries, and considering cumulative pressures) to assess and implement population level measures as an interim objective, where the ultimate aim of ASCOBANS, the Agreement on the Conservation of Small Cetaceans of the Baltic, North East Atlantic, Irish and North Seas (discussed in more detail below), is to reduce the number of such deaths to zero.

Increasing scientific literature demonstrates a need to manage human activities not only to maintain cetacean populations, but also to minimize welfare impacts on individual animals. Population level effects may take a long time to manifest or to be determined, if at all [for example, see (42)]. For those individuals that survive bycatch, but escape injured, the impact on their long-term welfare also has the potential to influence population level processes. Thus, estimation of “sustainability” based only on recorded or estimated deaths, without the consideration of sub-lethal welfare impacts across population level processes are inadequate. Whereas, animal welfare metrics can be observed in the short-term, thus enabling problems to be addressed more rapidly (43).

A broader and more ethical approach that tackles bycatch wherever it is known to occur, placing the highest priority on the fisheries with the largest bycatch, may be more effective from a welfare perspective, rather than only relating bycatch to population size and assessing whether it is “sustainable” before taking action (43). Improving measures to understand and reduce population level concerns would also reduce the number of individuals that suffer. Similarly, actions to optimize welfare can enhance conservation outcomes (44). A more balanced approach, where equal consideration is given to welfare and conservation, would comply with the emergent, and well-reasoned rational of “compassionate conservation” (45, 46).

The Treaty of Amsterdam contains a Protocol introducing legal obligations within the EU Treaty for parties to consider animal welfare in key areas of law and policy, recognizing the status of some species as “sentient beings” (47). As a result, there is a legal mandate and obligation to protect the welfare of sentient animals. In addition, it is asserted in the protocol that wild animals have intrinsic value. This highlights that while EU nations have a legal and moral imperative to address conservation issues caused by anthropogenic pressures, similarly there is also a legal and moral imperative to address animal welfare issues for sentient animals that arise as the result of anthropogenic pressures such as bycatch (48). Measures for protecting the welfare of sentient animals should be focused on optimally addressing animal needs for a particular set of circumstances by using animal-based measures based on the animal's perspective (49).

Considering the animal's perspective, here, we review the existing, compelling evidence of the extent of welfare impacts of cetacean bycatch globally, progress made toward implementation of welfare considerations in bycatch reduction, the welfare implications of bycatch mitigation strategies with a particular focus on the situation as it stands in European waters and case studies for two North East Atlantic cetaceans that face entanglement: common dolphins and minke whales.

## Welfare impacts associated with bycatch

To examine the question of whether traditional bycatch management practices that focus on “sustainability” need to be improved to include consideration of animal welfare, here we consider the fishing gear involved, the process of capture and the types of injuries sustained in bycatch and entanglement, the longer term sub-lethal impacts for those that escape or are released and the wider social impacts upon conspecifics (2, 50–52). Some pertinent cases are summarized here.

### Times to death or release from gear

The suffering of an odontocete captured in fishing gear is more likely to occur over a period of minutes or possibly hours. Porpoises can become enclosed rather than entangled and can still surface to breathe (such as in pound nets, herring weirs) (53) so might be trapped for longer, and can usually be released without apparent injuries. Baleen whale entanglements in fishing gear have been recorded to occur over much longer time periods. For example, the “very slow and likely extremely debilitating demise of the North Atlantic right whale averages 6 months, but there are cases that persist for multiple years” (5). Prolonged entanglement in fishing gear negatively affects the health and welfare of individual animals and can also lead to population level effects including reduced fecundity and survival (54).

### Assessment of injuries sustained

“There are clear differences in the types and degree of injuries received by bycaught cetaceans” (50), varying with species and with age (8). Pathological data for odontocetes indicate that the majority of bycaught cetaceans asphyxiate in the nets (8) or may drown. Before death, escape or release, injuries occur during interaction with the gear itself, through interactions with bycatch reduction devices or when the individual is hauled on board the fishing vessel (55).

Long-line fisheries can lead to entanglement of odontocetes and baleen whales and to injuries that result from depredation that include getting attached by one or more parts of the body to a baited hook (hooking) and entanglement in the fishing line. “Hooking is the result of a marine mammal being unable to dislodge itself from the hook, and the animal may remain attached to longline gear or break free, often with the hook still lodged in its mouth or other body part” (56).

Welfare assessment of stranded individuals has been studied directly resulting from fisheries in UK waters. “Post-mortem of 182 cetaceans stranded in the UK [comprising 97 harbor porpoise, 80 common dolphin, three striped dolphin, one Risso's dolphin (*Griseus grampus*) and one minke whale] from 1999 to 2005 found evidence of complex entanglements involving multiple parts of the body. External injuries included amputations (from entanglement or being cut free), broken maxillae, mandibles and/or teeth and internal injuries consisting of organ congestion, muscle tears and hemorrhaging (either from the gear or from the cetacean struggling)” (8).

Welfare assessment of free-swimming individuals demonstrated a high prevalence of injuries photographically in white-beaked dolphins (*Lagenorhynchus albirostris*) off the coast of Northumberland in the North Sea and off the coast of Iceland, from fisheries interactions and vessel strikes (57, 58).

### Longer term health responses

Non-lethal entanglement in fishing gear is sufficiently stressful to cause both a behavioral and physiological stress response in baleen whales (59). Fecal glucocorticoid studies have shown markedly elevated stress hormone levels in a severely entangled right whale (60), the relationships between entanglement stress and metabolic rate are complex. Long-term stress from being chronically wrapped in gear may explain why examined whales were unable to fight off the initial insult of infected gear lacerations, most likely leading to their demise (59). Visual health assessment of North Atlantic right whales using photographs demonstrated that stress responses existed that may have impacted health and fecundity even after the gear is no longer attached (61). Ultimately entanglements can lead to eventual lethal trauma through a drawn-out cumulative loss of body condition and constriction of body parts, with or without secondary infection, with expected extreme pain associated (5). Entanglements of baleen whales that eventually lead to death after a long period of suffering are, arguably, one of the worst forms of human-caused mortality in any wild animal (59).

High levels of stress are anticipated during capture and the physical and psychological stress and injuries for individuals that escape may cause prolonged suffering and/or subsequent mortality (51). Documented effects for those that escape or are released from fishing gear include behavioral alterations, physiological and energetic costs, such as associated reductions in feeding, growth, or reproduction (i.e., individual fitness) (51), potentially leading to reduced long-term survival. The full impact on an individuals' welfare and the extent to which this may affect mortality, life history events, and key biotic interactions and processes within the environment (62) are less well-known and so rarely, if ever, taken into consideration in sustainability analyses.

### Wider reaching impacts on conspecifics

We are beginning to understand the implications that bycatch has for conspecifics. Due to the highly social nature of many odontocetes, survival and reproductive success can depend on social cohesion and organization, and the effects of social disruption caused by bycatch mortalities may go beyond the dynamics of individual removals and impede population recovery (63, 64). Wade et al. (63) suggest that the social and behavioral traits of some odontocetes may contribute to a lack of resilience in some populations, specifically where survival and reproductive success may depend on: (a) social cohesion and organization, (b) mutual defense against predators and possible alloparental care, (c) inter-generational transfer of “knowledge,” and (d) leadership by older individuals.

One of the longest running and perhaps most informative studies of sub-lethal impacts resulting from fisheries interactions is from the Eastern Tropical Pacific (ETP). Despite a dramatic decrease in the number of northeastern offshore spotted dolphins (*Stenella attenuata attenuata*) and eastern spinner dolphins (*S. longirostris*) bycaught in this fishery, from more than six million to fewer than 1000 dolphins per year, the populations are not showing signs of recovery (65, 66). The rate of calf production has also been declining since the 1980s (67). Hypotheses to explain the lack of recovery (66) have included under reporting of kills by observers, cryptic effects of the fishery undetectable by observers, such as stress induced abortion, or the separation of mothers and calves (68). Permanently separated dependent calves may then represent unobserved mortality events which are a significant welfare concern since un-weaned calves may die of starvation following orphaning. This may partially explain the lack of recovery of depleted ETP dolphin populations (69) where, in the case of mothers dying, a calf or dependent juvenile must be assumed to become a secondary victim (68). There is also some evidence that setting nets on dolphins can result in miscarriage in pregnant females (70). It is plausible that the chase and encircling of the dolphins has hindered or prevented recovery in these populations, groups of individuals that show complex social structure [(63); Butterworth et al. in preparation^3^].

Observations of a bottlenose dolphin calf temporarily entangled in monofilament line showed immediate alterations in the behavior of the mother and calf, as well as conspecifics (71). A similar pattern of seemingly social avoidance by conspecifics following entanglement has occurred on a number of occasions [(72) and references therein], where the costs of entanglement (e.g., infection, injury, energetic costs, inability to forage), are likely exacerbated. As well as causing distress to surviving family or social group members, the loss of key individuals may lead to the loss of important social knowledge (73). Frère et al. (74) examined genetic and social effects on female calving success (a partial measure of fitness) in bottlenose dolphins. They determined that both genetic and social factors contribute to variation in individual fitness related to female calving success. They posit that the influence of social relationships between females is consistent with either the social transmission of reproductive prowess, or with a type of homophily in which females with calves associate with other females with calves [as suggested by (75)].

## Progress toward implementation of welfare considerations in european bycatch reduction

The key regional scientific, legislative and policy mechanisms used to engage, collect and share data in European waters are reviewed here.

### Post-mortem analysis to understand welfare impacts

Post-mortem analysis of stranded cetaceans and data from bodies collected from fishing boats provides the best opportunities for welfare assessment. Some injuries, such as external signs of acute entanglement, red or bulging eyes and multi-organ congestion, can be reliably used for bycatch diagnosis (76) and indicates the extreme conditions under which these cetaceans die. Strandings data provide an indication of the range of species that have been bycaught. For example, in UK waters, in addition to those species observed as part of the on-board bycatch observation scheme, stranded bycaught species have included minke whales, as well as long-finned pilot (*Globicephala melas*) and humpback whales. Collection of carcasses enables assessment of welfare implications and strandings can also provide an early indication of a newly emerging issue at sea, including bycatch from a novel source.

#### European union legislation

Existing European Union legislation includes no explicit provisions for the protection of cetacean welfare from incidental bycatch (8). The European Council Directive 92/43/EEC (1992) on the Conservation of Natural Habitats and wild fauna and flora, the Habitats Directive [Article 12(4)], requires “*Member States shall establish a system to monitor the incidental capture and killing of the animal species listed in Annex IV (a)*” (which includes cetaceans) and secondly, “*In the light of the information gathered, Member States shall take further research or conservation measures as required to ensure that incidental capture and killing does not have a significant negative impact on the species concerned*.” Despite the clear requirement of the Directive, measures to implement it remain largely inadequate.

After more than a decade of implementation of EC Regulation 812/2004 on the incidental catches of cetaceans, compliance is inadequate and subsequently, levels of dolphin, porpoise and whale bycatch in static and mobile fishing gear are not clearly known. The existing EU Regulation 812/2004 is not entirely fit for purpose and doesn't focus on appropriate fisheries to adequately assess bycatch in EU waters. Further, 15 out of 17 Member States implementation of the Regulation has generally been poor or moderate (77).

Despite plenty of evidence in recent years to demonstrate the flaws in Regulation 812, based on this and other EU scientific reports (27, 78–83), technical conservation measures drafted by the EU Commission (84) in March 2016 [file 2016/0074 (COD)] do not significantly improve them. The proposal incorporates the main mitigation and monitoring requirements contained in Council Regulation (EC) 812/2004 and a geographic extension of the mandatory use of acoustic deterrent devices (ADDs) to all sea basins (to include full coverage in the Baltic Sea and in South Western Waters and the West of Scotland [DG (85)]. This file was considered by the European Council and Parliament, reports have been produced from each and discussions are currently in trilogue negotiations between them. Although amendments to account for and improve welfare standards in bycatch were put forward by a Member of the European Parliament Committee on Fisheries as amendments to the Fisheries Committee (86), these were rejected in a vote of the Committee of Fisheries. The amendments included “*ensure that the impacts of fishing on the welfare of animals are minimised and where possible eliminated”* (AM329)*, “The use of innovative fishing gears shall only be permitted if those assessments indicate that their use will not lead to negative impacts on animal welfare, sensitive habitats and non-target species*” (AM543) and “*Fish and other marine animals are sentient beings, and the Union's fisheries policies shall therefore pay full regard to the welfare requirements of these animals”* (AM251). More generally, under the Data Collection Framework, data on incidental bycatch of all birds, mammals and reptiles and fish protected under Union legislation and international agreements, including absence in the catch, needs to be collected during scientific observer trips on fishing ships or by the fishers themselves through logbooks. Where data collected during observer trips are not considered adequate to provide sufficient data on incidental bycatch for end-user needs, other methodologies need to be implemented by Member States. The selection of these methodologies shall be coordinated at marine region level and be based on end-user needs (DG (85)].

### ASCOBANS

ASCOBANS maintains the goal of reducing bycatch toward zero, an ambition that is motivated by welfare concerns. ASCOBANS produces species action plans that contribute to achieving this aim, including for harbor porpoise in the Western Baltic, the Belt and the Kattegat Sea (87), Baltic Sea (23) and North Sea (88) and a conservation plan for common dolphin (ASCOBANS, in preparation). Bycatch has been identified as the highest priority for action. An ASCOBANS Bycatch Working Group exists and a number of bycatch work streams and associated workshops have taken place^4^.

Strandings remain on the agenda at ASCOBANS, where Resolution No.10 on Small Cetacean Stranding Response was passed at the most recent Meeting of the Parties (89). The Resolution calls on Parties to establish and fund strandings networks, including to conduct post-mortem examinations and to share data.

### International whaling commission

The IWC has a long history of dealing with cetacean welfare issues. The IWC Welfare Workshop (3) recommended a high priority be placed on developing effective entanglement mitigation and prevention measures, and until such time as that is developed, continue support for the palliative care offered by further developing the Global Whale Entanglement Response Network and database. The Workshop further recommended that a more detailed consideration is carried out on the implications of entanglement and bycatch for small cetaceans.

In evaluating the impacts on animal welfare, assessment should consider both the severity and the duration of negative health and stress measurements (44). The more recent IWC Welfare Workshop (3) recommended monitoring of wound healing, wound progression, and time to death in cetaceans in the wild that have incurred vessel-strike or entanglement injuries, in order to provide greater understanding of the welfare implications for individuals (3).

In 2016, an IWC cetacean strandings workshop concluded that an international Strandings Network, involving experts from a number of different countries, should be established. It could help to standardize data and mitigate impacts from man-made sources^5^. IWC has now appointed a strandings coordinator.

Beyond large whale entanglements IWC also recognizes the severity of bycatch impacts on cetaceans and has now established a Bycatch Mitigation Initiative.

## Welfare implications of bycatch mitigation strategies

Marine mammal bycatch mitigation strategies encompass both the prevention and reduction of incidence and severity, and the first priority of any bycatch management strategy should be the prevention of entanglement or bycatch (54).

Recognizing the scale of bycatch, mitigation strategies have been developed in a number of fisheries. Mitigation options include management measures such as spatial or temporal management of fishing, and technical solutions including: modifying the gear, either to make it more visible (for example using acoustic devices) or reducing the likelihood of entanglement once a cetacean makes contact with the gear, or reducing the severity of entanglement (e.g., weaker line). Existing mitigation options have been reviewed in detail (53).

Banning or restricting fishing (including the use of closed areas or closed seasons) in areas used by cetaceans can be effective if properly enforced. The most generally effective mitigation of cetacean bycatch and entanglement is a reduction in effort, starting with those fisheries that have the largest bycatch (53). Reducing effort and bycatch would clearly also reduce welfare impacts. If reducing effort is not deemed possible then modifying gear or replacing gear types to reduce risk of contact or entanglement are the main strategies known to reduce risk of bycatch (53) and so would also reduce welfare impacts, as would minimizing gear loss and “wet” storage of gear at sea when not in use. The most promising solutions lie with the development of alternative gear to replace current fishing methods such as gillnets (53).

Some mitigation measures reduce the numbers of individuals killed but have additional impacts that can affect welfare. The use of active acoustic devices (such as pingers) has been demonstrated to successfully modify the behavior of some dolphins, porpoises and small whales to reduce the frequency of their interactions with gillnet fisheries (90). Pingers on drift nets successfully eliminated beaked whale bycatch in the Californian drift gill net fishery (91), where the species previously caught included Cuvier's beaked whales (*Ziphius cavirostris*), Hubb's beaked whales (*Mesoplodon carlhubbsi*), Stejneger's beaked whale (*M. stejnegeri*), Baird's beaked whale (*Berardius bairdii*), as well as unidentified Mesoplodon and ziphiid species. However, pingers may lead to displacement from important habitats, with unknown welfare implications or, theoretically if sources levels are loud enough, could potentially cause auditory damage (92).

There are also welfare concerns associated with some odontocete bycatch mitigation efforts that involve the use of trap doors, escape hatches and exclusion grids, that might be used to allow individuals to escape from a large net once a dolphin has entered. Behaviors exhibited by a number of species that interacted with a bycatch reduction device in a trawl net included the animal becoming caught in the mesh by fins, head or tail; the tail being caught or stuck in the exclusion grid; the animal remaining in the net after a stressful interaction with the grid or mesh; the animal continuing to move and remaining in the net motionless after stressful interaction with grid or mesh; and finally, of the animal being assumed dead, when potentially still alive (93).

Adaptive management principles would enable scientifically credible monitoring programmes to measure key performance indicators (46), enabling an understanding of the consequences of management decisions to make the appropriate decisions accordingly. As an example, van Beest et al. (94) found that a mix of pingers and spatial restrictions had the best effect on reducing bycatch and disturbance.

Case studies are provided here of the different issues faced by bycaught common dolphins and entangled minke whales in European waters and the associated welfare impacts.

## Case study: common dolphin bycatch in european waters

Bycatch has been identified as the greatest anthropogenic threat to common dolphins (26, 95–97) and at levels such that it may be having a population level effect in European waters (83). The most recent assessment (80) of the conservation status for the European Atlantic common dolphin population under Article 17 of the Habitats Directive was “Unfavourable-Inadequate”. From a welfare perspective, a greater number of individuals bycaught from a large population is a greater concern than a smaller bycatch from an endangered population. Bycatch estimates from strandings data and observer programmes demonstrate that, whilst the figures vary from 1 year to the next, thousands of common dolphins have been bycaught in European fisheries each year over the last three decades (26, 96).

The highest levels of common dolphin bycatch were observed in the nets of mobile pelagic trawl fisheries (especially pair-trawls, where two boats fish with a net stretched out between them), with lower levels observed in static gillnet fisheries, although these may be equally significant as they may result in similar levels of total bycatch due to higher fishing effort by static net fisheries [ASCOBANS, in preparation; (83)]. Many European countries operate fishing gear in the region. A number of fisheries are not adequately monitored for bycatch, despite clear indications that bycatch is occurring, including in the offshore fleet such as pelagic freezer trawlers, high vertical opening trawlers and some bottom set gill nets (26). The full extent of bycatch in European waters remains uncertain as monitoring occurs on a very small percentage of part of the fleet and dolphins bycaught further offshore may be less likely to come ashore, be reported and subsequently post-mortemed.

### Injuries sustained

Data from on-board fishing vessels and stranded individuals provide important welfare information about impacts [for example, (8)]. More than 41% of common dolphins suffered broken beaks and others had broken maxillae or mandibles (24.2%) and/or broken teeth (8). Broken beaks are thought to result from capture in mobile fishing gear, whilst finer net marks are a more obvious sign of capture in static fishing nets. The tail, pectoral fins and head/beak were more likely to have net marks than the dorsal fin. Amputations were noted frequently in common dolphins and harbor porpoises, but it was unclear whether these were due to entanglement in nets or from being cut free (8).

In general, a large proportion of bycaught cetaceans had generalized organ congestion (liver, kidneys, spleen, and adrenal glands) caused by reduced blood flow. Internal injuries can be inflicted by the fishing equipment and also by the cetacean struggling to free itself. Soulsbury et al. (8) note that since entrapped cetaceans typically make powerful dorso-ventral and lateral movements, these probably cause the hemorrhaging and tears in the longissimus dorsi muscle, which is the primary swimming muscle. Similarly, because the pectoral fins frequently become entangled, such movements will cause muscle tears and hemorrhaging in the peri- and subscapular areas, and torsion of the body leads to internal hemorrhaging of the thoracic rete mirabile.

### Potential solutions

Sea bass pair-trawling, other pelagic vessels and set-nets result in common dolphins deaths in large numbers each winter. Due to poor sea bass stocks, a ban has been in place on the pelagic trawl fishery for sea bass in the English Channel, Celtic Sea, Irish Sea and southern North Sea during February and March, since 2015. High levels of common dolphin bycatch were still documented in these months in the winters of 2016 and 2017. Therefore, it is necessary to conduct monitoring to understand in more detail which other parts of the fishing industry, in particular the offshore fleet that is largely unmonitored such as pelagic freezer trawlers, high vertical opening trawlers and bottom set gill nets, might also have dolphin bycatch.

Better monitoring is also required on a broader range of vessel sizes within the fleet, including vessels smaller than 15 m. Monitoring should be conducted using independent on-board observers or tamper proof video cameras (remote electronic monitoring) to understand which elements of the fleet require the implementation of mitigation measures. Compulsory reporting of all bycatch incidents by fishermen should be an additional requirement, recognizing that these data are necessary and can be used sensitively to inform future management.

In addition, simple changes to fishing practices might reduce bycatch. For example, fishing only during daylight hours and fishing in waters over a certain depth have been shown to prevent common dolphin bycatch in Galicia, Northwest Spain (95). All gillnet operators in the Coorong Zone in South Australia must cease fishing and move fishing operations at least five nautical miles away if there is any dolphin bycatch. The purpose of this measure is to encourage fishers to adapt their fishing practices on the water and reduce the risk of further dolphin bycatch by immediately moving away from the location of a dolphin bycatch event (98).

Evidence suggests that common dolphin bycatch may have decreased when loud pingers were voluntarily introduced on some nets in parts of the UK sea bass pair-trawl fleet (99). Trap doors have been trailed in some trawl fisheries to reduce common dolphin deaths. The welfare concerns associated with the use of both these mitigation options were discussed above and require consideration in developing a suitable solution to common dolphin bycatch.

Development of a multi-pronged approach is required to reduce bycatch—such as requiring electronic monitoring as well as reporting bycatch incidents. Mitigation measures might include a focus on implementation of benign mitigation measures, such as moving away when dolphins are spotted and not operating at night (96). Pingers might be trialed, and tested for effectiveness, for individuals missed during a scan from the bridge of the boat or for those that approach the vessel during fishing operations. Trap doors should only be implemented with adequate trials to monitor potential welfare impacts on individuals that become trapped but are able to escape through the trap door.

Efforts will need to be collaborative across the range of nations that fish in these waters and so the ASCOBANS Common Dolphin Action Plan (ASCOBANS, in preparation) may be an important starting point if countries invest.

## Case study: minke whale entanglement in scottish waters

About 50% of post-mortemed minke whales in Scottish waters have been diagnosed as having died due to entanglement in creel lines and other ropes (100) As many as 17.7% of identified minke whales observed at sea in the Hebrides show some evidence of previous entanglement between 2009 and 2011 (101).

A wider analysis between 1990 and 2010 demonstrated that the head is the body region most commonly found with scars indicative of entanglement, suggesting that minke whales may become entangled in fishing gear whilst feeding (101). Minke whale entanglements have a higher fatality rate and are less likely to be noted ante-mortem than humpbacks and other larger baleen whales because minke whales are less powerful swimmers and so may be less likely to reach the surface to breathe whilst entangled (102). Minke whales tend to become tethered in pot lines, rather than picking up and carrying the gear. Katona et al. (103) report a single observation of a minke whale in the North Atlantic surviving submerged for 17 min as it was being freed from a fish weir. Leaper et al. (104) discuss times to death, where the trauma associated with prolonged submersion until death in this species. Pathological changes have been noted in cetacean tissues associated with death from asphyxiation (105–109) and such signs are indicative of physiological stress and a potentially protracted dying process (104).

A cetacean entangled underwater is in a potentially terminal forced dive situation. The whale may adopt one of two strategies: induce a rapid and profound dive response (though it is difficult to identify an adaptive explanation for such behavior if the whale has been entangled and potentially perceives an opportunity to break free); or start to struggle. There is evidence of the latter behavior from tissue damage to entangled marine mammals (104). If the whale struggles frantically to free itself then this effort will require an increased oxygen supply to muscles. Whereas, a whale that does not struggle may show the accentuated bradycardia seen in forced submergence (104).

### Potential solutions

Adoption of ropes with lower breaking strengths (of 1,700 lbs or less) could reduce the number of life-threatening entanglements for large whales by at least 72%, and still be strong enough to withstand the routine forces involved in many fishing operations (102). Measures that might work for humpback whales if used throughout the fishery may not be useful for much smaller and lighter minke whales. Lines that are weak enough for minke whales to escape may be possible in some shallow, sheltered areas, where the pulling load is less when gear is being hauled back on-board the fishing vessel. Nevertheless, reducing the amount of fishing rope in the water column is likely to be the most successful entanglement prevention strategy. Rope-less technologies are being developed that may help reduce entanglements in the future if widely implemented.

In summary, the welfare issues identified for both common dolphins and minke whales are likely to be severe, and indicate that the welfare of all bycaught cetaceans is often very poor. Better monitoring is required to understand the extent of entanglement for both species. Tried and tested mitigation measures to reduce the welfare impacts for both species include reducing the amount of fishing gear in the water. Technical mitigation measures available for common dolphin bycatch in mobile gear have associated welfare issues that are yet to be resolved and reliable mitigation measures for whale entanglements in fishing rope are still under development.

## Conclusions and recommendations

It is the authors' contention that policy decisions surrounding fishing and bycatch do not adequately consider the variety of welfare impacts of bycatch on cetaceans. Animal welfare considerations should be an integral part of conservation decision-making from both a robust scientific and an ethical perspective. To address the ubiquitous and considerable welfare issues arising from bycatch and entanglement, more robust and transparent management systems are urgently required, with the aim to better document and work toward eliminating bycatch altogether. As a result of the demonstrable suffering resulting from bycatch and entanglement, and in line with legislative mandates across the EU, animal welfare considerations should become a central tenant to fisheries policy-decision making. Activities that put animal interests at risk should be independently regulated. Changing the government's approach to welfare is an essential precondition to achieving legitimate and effective standards of animal protection (110).

Marine mammal bycatch mitigation strategies encompass both the prevention and reduction of incidence and severity, and the first priority of any bycatch management strategy should be the prevention of entanglement or bycatch (54).

A number of different stakeholders have valuable roles in eliminating welfare impacts. Fishers themselves can be encouraged or required to document and report entanglements, accommodate independent observers on-board or use electronic monitoring to collect and bring bycaught individuals to harbor for post-mortem examination and to implement bycatch solutions. Researchers have the role of analyzing post-mortem data of bycaught individuals, as well as monitoring population health of live individuals (for example, using photo-identification to understand scarring) and developing sophisticated measures for welfare assessment. Managers have the role to legislate for improvements in fisheries and bycatch data collection and prevention. Conservation and welfare groups can raise awareness amongst the public about their consumer choices and amongst politicians and decision makers to improve legislative measures to reduce bycatch and concurrently to improve welfare. Engineers can develop fishing techniques that do not have associated bycatch. Effective bycatch mitigation will require coordinated action by the range of stakeholders and actors to develop a combination of changes in fishing practices, modification of fishing effort, technological gear fixes and international agreements that, together, can monitor and mitigate bycatch (111).

Explicit policy decisions and rigorous implementation are urgently needed to bridge the gap between our poor understanding and the reality of what is happening at sea (12). Political motivation and transparent consideration of the sub-lethal costs of bycatch and entanglement in decision making are essential.

Bycatch is not intentional, but neither can it be regarded as entirely accidental and many fishermen are involved in strategies to reduce the incidental capture of cetaceans. The approaches required will often be fishery specific, and all solutions are dependent on positive relationships and involvement with fishermen. Participation of fishermen in the management process is necessary (112), bycatch reduction approaches can be implemented successfully from the bottom-up in the hands of fishermen (113). Incentive-based management measures are likely to be most effective to engage fishermen.

There is a great need for effective mitigation measures to address bycatch of marine mammals, including in gill-net fisheries (114) and there also remains an urgent need for better entanglement avoidance and disentanglement initiatives for baleen whales.

Where mitigation methods implemented result in welfare impacts, such impacts require monitoring to understand and evaluate the consequences. The sub-lethal effects of injuries caused as a result of bycatch and stress on fitness and the length of time to asphyxiation are not as well-understood as they might be. The social implications of individuals dying are a further area that would benefit from better knowledge. However, a higher priority would be to better fund research into effective methods to stop bycatch from occurring. In addition, information about its scale requires wider publicity and better public awareness.

To reduce suffering as a result of bycatch requires, a transparent, multi-taxa approach, a framework and timeframe to reduce bycatch, incentives for fishermen: encouraging implementation of best practice: i.e., reporting all incidences, as well as application of electronic monitoring and adaptive at-sea management.

Market-based mechanisms should include retailers and suppliers working with fisheries to improve transparency of practices and governance. As a component of this, certification schemes should include the mortality and welfare considerations of bycatch in their assessments of fisheries and clear labeling of the resulting fish products. A major effort to educate seafood consumers as to the chronic and widespread welfare concerns that marine mammal bycatch and entanglements represent would help achieve their mitigation through consumer pressure.

The MSC is undertaking a review of its Fisheries Standard in 2018 and 2019. A review of MSC's requirements for assessing the impact of fisheries on endangered, threatened and protected (ETP) species requirements will form a major part of the Fisheries Standard review, where the MSC recognizes the importance of providing robust protection for these species, and the need to address the cumulative impacts of a fishery upon them (115). Conservation and welfare groups efforts are increasingly focused on supermarkets, who have a powerful role in sourcing seafood and so can influence MSC and other “ecolabels” to continually improve their standards to account for bycatch more transparently and in a more consistent way, in their assessment and accreditation processes.

We argue that current policy decisions surrounding fishing do not adequately consider cetacean bycatch, including the welfare implications of bycatch. There are welfare issues in all bycatch situations and suffering cannot be reduced without preventing bycatch. The well-documented welfare implications of marine mammal bycatch provide a strong argument for zero-tolerance on cetacean bycatch and make a compelling case for immediate action to reduce bycatch toward zero. Uncertainties around the true magnitude of bycatch should not delay management decisions, icluding where bycatch is considered “sustainable.”

To deal with these welfare issues, a clear, timelimited, and effective strategy is needed to identify the steps that are required by all fisheries to reduce bycatch toward zero (12) and this should include welfare specific legislation for marine species, as already exists for terrestrial mammals. There is strong scientific, ethical, consumer, and political mandate for animal welfare implications resulting from bycatch to become an integral part of fisheries policy and conservation decision-making.

## Author contributions

SD reviewed the literature and wrote the first draft manuscript. PB provided detailed input to draft manuscript. Both authors provided feedback to reviewers comments and proofread the manuscript.

### Conflict of interest statement

The authors declare that the research was conducted in the absence of any commercial or financial relationships that could be construed as a potential conflict of interest.

## Footnotes

^1^Independent. (2006). Dolphin Friendly Tuna? Don't believe it. 12^th^ October 2006. https://www.independent.co.uk/environment/dolphin-friendly-tuna-dont-believe-it-419728.html; National Geographic. (2014). The ABCs of Ecosystem-Based Fisheries Management—Part III. Reducing and minimizing bycatch. 14^th^ May 2014. https://blog.nationalgeographic.org/2014/05/14/the-abcs-of-ecosystem-based-fisheries-management-part-iii/; Blue Planet Society. (2018). Decades of needless dolphin deaths must end. 10^th^ March 2018. http://blueplanetsociety.org/2018/03/decades-needless-dolphin-deaths-must-end/

^2^Forbes. (2015). 'Dolphin Safe' Labels On Canned Tuna Are A Fraud. 29^th^ April 2015. https://www.forbes.com/sites/realspin/2015/04/29/dolphin-safe-labels-on-canned-tuna-are-a-fraud/#23395d71295e; Telegraph. (2018). Misleading 'dolphin friendly' claims could be illegal. 18^th^ June 2018. https://www.telegraph.co.uk/foodanddrink/8250917/Misleading-dolphin-friendly-claims-could-be-illegal.html.

^3^Vail Philippa Brakes CS, Reiss D, Butterworth A. Potential welfare impacts of chase and capture of small cetaceans during drive hunts in japan (in review).

^4^http://www.ascobans.org/fr/species/threats/bycatch.

^5^https://iwc.int/strandings.

## References

[1] Read AJ Drinker P Northridge S. Bycatch of marine mammals in U.S. and Global Fisheries. Conserv. Biol. (2006) 20:163–9. 10.1111/j.1523-1739.2006.00338.x 16909669

[2] Dolman SJ Moore MJ Chapter 4. Welfare implications of cetacean bycatch and entanglements. In: Butterworth A, editor. Animal Welfare. Cham: Springer (2017).

[3] IWC Report of the Workshop to Support the IWC's Consideration of Non-Hunting Related Aspects of Cetacean Welfare. (2017). Available online at: https://iwc.int/iwc-workshop-on-welfare-may-2016 (Accessed May11, 2018).

[4] Fraser D Weary DM Pajor EA Milligan BM A scientific concept of animal welfare that reflects ethical concerns. Anim. Welf . (1997) 6:187–205.

[5] Moore MJ van der Hoop JM The painful side of trap and fixed net fisheries: chronic entanglement of large whales. J. Mar. Biol. (2012) 2012:230653 10.1155/2012/230653

[6] Moore M Welfare of whales bycaught in fishing gear or struck by vessels. Anim. Welf . (2013) 22:117–21. 10.1007/978-3-319-46994-2_4

[7] Porter DG. Ethical scores for animal experiments. Nature (1992) 356:101–2. 10.1038/356101a0 1545854

[8] Soulsbury CD Iossa G Harris S The Animal Welfare Implications of Cetacean Deaths in Fisheries. A University of Bristol report to the Whale and Dolphin Conservation Society (WDC). (2008).

[9] OIE Terrestrial Animal Health Code. World Organisation for Animal Health. Vol. 1. (2017). Available online at: http://www.oie.int/standard-setting/terrestrial-code/access-online/ (Accessed June15, 2018).

[10] Reeves RR McClellan K Werner TB Marine mammal bycatch in gillnet and other entangling net fisheries, 1990 to 2011. End. Sp. Res. (2013) 20:71–97. 10.3354/esr00481

[11] Williams R Burgess MG Ashe E Gaines SG Reeves RR. U.S. seafood import restriction presents opportunity and risk. Science (2015) 354:1372–4. 10.1126/science.aai8222 27980169

[12] Dolman SJ Baulch S Read F Ritter F Evans P Towards an EU action plan on Cetacean bycatch. Mar. Pol. (2017) 72:67–75. 10.1016/j.marpol.2016.06.020

[13] Norden W Athearn K McNaught D Larkin S Teisl M Assessing the Impact of MSC Certification on Management and conservation in the New Zealand Hoki and Orange Roughy fisheries. Report to the David and Lucile Packard Foundation – Project # 2008–32492. (2011).

[14] Christian C Ainley D Bailey M Dayton P Hocevar J LeVine M A review of formal objections to Marine Stewardship Council fisheries Certifications. Biol Conserv. (2013) 161:10–7. 10.1016/j.biocon.2013.01.002

[15] Wang R Analyzing Bycatch Mitigation in the MSC-Certified Canadian Northwest Atlantic longline swordfish fishery. Thesis submitted for the degree of Master of Marine Management at Dalhousie University, Halifax, Nova Scotia. (2013).

[16] Gutierrez A Thornton TF Can consumers understand sustainability through seafood eco-labels? A U.S. and UK Case Study. Sustain (2014) 6:8195–217. 10.3390/su6118195

[17] Wade PR Watters GM Gerrodette T Reilly SB Depletion of spotted and spinner dolphins in the eastern tropical Pacific: modeling hypotheses for their lack of recovery. Mar Ecol Prog Ser. (2007) 343:1–14. 10.3354/meps07069

[18] Vinthner M Bycatches of harbour porpoises in Danish set-net fisheries. J Cetacean Res Manage. (1999) 1:123–35.

[19] Bjørge A Skern-Mauritzen M Rossman MC Estimated bycatch of harbour porpoise (*Phocoena phocoena*) in two coastal gillnet fisheries in Norway, 2006–2008. Mitigation and implications for conservation. Biol. Conserv. (2013) 161:164–73. 10.1016/j.biocon.2013.03.009

[20] ASCOBANS NSSG Report of the 4th Meeting of the ASCOBANS Steering Group for the Conservation Plan for the Harbour Porpoise in the North Sea. ASCOBANS AC22/Doc. 2.2. (Accessed July 30, 2015) (2014).

[21] Northridge S Kingston A Thomas L Annual Report on the Implementation of Council Regulation (EC) No 812/2004 during 2014, UK. St Andrews University (2015).

[22] Northridge S Kingston A Thomas L Annual Report on the Implementation of Council Regulation (EC) No 812/2004. SMRU Commissioned Report for Defra (2016).

[23] ASCOBANS Recovery Plan for Baltic Harbour Porpoises. (2016). Available online at: http://www.ascobans.org/sites/default/files/document/ASCOBANS_JastarniaPlan_MOP8.pdf (Accessed March 12, 2018).

[24] Read FL Understanding Cetacean and Fisheries Interactions in the North-West Iberian Peninsula. Ph.D. thesis, University of Aberdeen (2016).

[25] ICES Working Group on Bycatch of Protected Species (WGBYC), 1–5 February 2016, ICES HQ. Copenhagen: ICES CM 2016/ACOM:27 (2016).

[26] Peltier H Authier M Deaville R Dabin W Jepson PD Van Canneyt O Small cetacean bycatch as estimated from stranding schemes: the common dolphin case in the northeast Atlantic. Environ. Sci. Policy (2016) 63:7–18. 10.1016/j.envsci.2016.05.004

[27] ICES Bycatch of Small Cetaceans and Other Marine Animals – Review of National Reports Under Council Regulation (EC) No. 812/2004 and Other Published Documents. (2015). Available online at: http://www.ices.dk/sites/pub/Publication%20Reports/Advice/2015/2015/Bycatch_of_PETS_Advice_2015.pdf

[28] Silvani L Gazo M Aguilar A Spanish driftnet fishing and incidental catches in the western Mediterranean. Biol Conserv. (1999) 90:79–85.

[29] Birkun AAJ Frantzis A Phocoena phocoena ssp. relicta. In: IUCN 2011. IUCN Red List of Threatened Species, Version 2011.2. Gland: IUCN (2008). Available online at: www.iucnredlist.org

[30] Vishnyakova K Gol'din P Seasonality of strandings and bycatch of harbour porpoises in the Sea of Azov: the effects of fisheries, weather conditions, and life history. ICES J Mar Sci. (2015) 72:981–91. 10.1093/icesjms/fsu192

[31] van der Hoop JM Moore MJ Fahlman A Bocconcelli A George C Jackson K Behavioral impacts of disentanglement of a right whale under sedation and the energetic cost of entanglement. Mar. Mam. Sci. (2013) 30:282–307. 10.1111/mms.12042

[32] Ryan C Leaper R Evans PGH Dyke K Robinson KP Haskins GN Entanglement: an Emerging Threat to Humpback Whales in Scottish Waters. Presented to the Scientific Committee Meeting of the International Whaling Commission, 2016, SC/66b/HIM/01. (2016).

[33] Lien J Entrapment of large cetaceans in passive inshore fishing gear in Newfoundland and Labrador (1979-1990). Rep. Int. Whal. Comm. (1994) 15:149–57.

[34] Wade PR Calculating limits to the allowable human-caused mortality of cetaceans and pinnipeds. Mar. Mam. Sci. (1998) 14:1–37. 10.1111/j.1748-7692.1998.tb00688.x

[35] Brandon JR Punt AE Moreno P Reeves RR Towards a tier system approach for calculating limits on human-caused mortality of marine mammals. ICES J. Mar. Sci. (2017) 74:877–87. 10.1093/icesjms/fsw202

[36] Punt AE Moreno P Brandon JR Mathews MA Conserving and recovering vulnerable marine species: a comprehensive evaluation of the US approach for marine mammals. J. Mar. Sci. (2018) 75:1813–31. 10.1093/icesjms/fsy049

[37] Berggren P Wade PR Carlström J Read AJ Potential limits to anthropogenic mortality for harbour porpoises in the Baltic Region. Biol. Conserv. (2002) 103:313–22. 10.1016/S0006-3207(01)00142-2

[38] Winship AJ Estimating the Impact of Bycatch and Calculating Bycatch Limits to Achieve Conservation Objectives as Applied to Harbour Porpoise in the North Sea. Ph.D. Thesis, University of St Andrews, Scotland. (2009).

[39] Scheidat M Leaper R van den Heuvel-Greve M Winship A Setting maximum mortality limits for harbour porpoises in Dutch waters to achieve conservation objectives. Open J. Mar. Sc. (2013) 3:133–9. 10.4236/ojms.2013.33014

[40] Robards MD Burns JJ Meek CL Watson A. Limitations of an optimum sustainable population or potential biological removal approach for conserving marine mammals: Pacific walrus case study. Environ. Manage. (2009) 91:57–66. 10.1016/j.jenvman.2009.08.016 19783356

[41] Smith Z Gilroy M Eisenson M Schnettler E Stefanski S Net Loss: the Killing of Marine Mammals in Foreign Fisheries. NRDC Report. 48 pages. (2014). Available online at: https://www.nrdc.org/sites/default/files/mammals-foreign-fisheries-report.pdf (Accessed June 18, 2018).

[42] Taylor BL Martinez M Gerrodette T Barlow J Hrovat YN Lessons from monitoring trends in abundance of marine mammals. Mar. Mam. Sci. (2007) 23:157–75. 10.1111/j.1748-7692.2006.00092.x

[43] Papastavrou V Leaper R Lavigne D Why management decisions involving marine mammals should include animal welfare. Mar. Pol. (2017) 79:19–24. 10.1016/j.marpol.2017.02.001

[44] IWC Report of the Whale Welfare and Ethics Workshop. Eden Project, Cornwall, United Kingdom on 22/23 March 2011. IWC/63/WKMandAWI 4. (2011). Available online at: https://awionline.org/sites/default/files/uploads/documents/ml-whalewelfareethicsworkshopreport-101111.pdf (Accessed March 9, 2018).

[45] Paquet PC Darimont CT Wildlife conservation and animal welfare: two sides of the same coin? Anim Welf. (2010) 19:177–90.

[46] Ramp D Bekoff M Compassion as a practical and evolved ethic for conservation. Bioscience (2015) 65:323–27. 10.1093/biosci/biu223

[47] Camm T Bowles D Animal welfare and the treaty of Rome - legal analysis of the protocol on animal welfare and welfare standards in the European Union. J Environ Law. (2000) 12:197–205. 10.1093/jel/12.2.197

[48] De Vere AJ Lilley MK Frick EE Anthropogenic impacts on the welfare of wild marine mammals. Aquat Mam. (2018) 44:150–80. 10.1578/AM.44.2.2018.150

[49] Miller DS Anthony R Golab G Assessing aquatic mammal welfare while assessing differing values and imperfect tradeoffs. Aquat. Mam. (2018) 44:116–41. 10.1578/AM.44.2.2018.116

[50] Jepson PD Barbieri M Barco SG Bernaldo de Quiros Y Bogomolni A Danil K Peracute underwater entrapment of pinnipeds and cetaceans. D Aquat Org. (2013) 103:229–64. 10.3354/dao02566

[51] Wilson SM Raby GD Burnett NJ Hinch SG Cooke SJ Looking beyond the mortality of bycatch: sublethal effects of incidental capture on marine animals. Biol Conserv. (2014) 171:61–72. 10.1016/j.biocon.2014.01.020

[52] Dolman SJ Asmutis-Silvia R Ryan C Whale entanglement – a 21st Century challenge in the ocean. In: Butterworth A, editor. Animal Welfare in a Changing World. Oxfordshire: CABI (2018).

[53] Leaper R Calderan S Review of Methods Used to Reduce Risks of Cetacean Bycatch and Entasnglements. Report submitted to Convention on Migratory Species, 12^th^ meeting of the Conference of the Parties. (2017). 29 p.

[54] FAO Expert Workshop on Means and Methods for Reducing Marine Mammal Mortality in Fishing and Aquaculture Operations. Rome, 20–23 March 2018. (2018).

[55] Kirkwood JK Bennett PM Jepson PD Kuiken T Simpson VR Baker JR. Entanglement in fishing gear and other causes of death in cetaceans stranded on the coasts of England and Wales. Vet Rec. (1997) 141:94–8. 9265709 10.1136/vr.141.4.94

[56] Werner TB Northridge S McClellan Press K Young N Mitigating bycatch and depredation of marine mammals in longline fisheries. ICES J Mar Sci. (2015) 72:1576–86. 10.1093/icesjms/fsv092

[57] Bertulli CG Cecchetti A Van Bressem MF Van Waerebeek K Skin disorders in common minke whales and white-beaked dolphins off Iceland, a photographic assessment. J. Mar Anim Ecol. (2012) 5:29–40.

[58] van Bressem M-F Burville B Sharpe M Berggren P van Waerebeek K Visual health assessment of white-beaked dolphins off the coast of Northumberland, North Sea, using underwater photography. Mar. Mam. Sci. (2018) 34:1119–33. 10.1111/mms.12501

[59] Cassoff RM Moore KM McLellan WA Barco SG Rotstein DS Moore MJ. Lethal entanglement in baleen whales. Dis Aquat Org. (2011) 96:175–85. 10.3354/dao02385 22132496

[60] Hunt KE Rolland RM Kraus SD Wasser SK. Analysis of fecal glucocorticoids in the North Atlantic right whale *(Eubalaena glacialis)*. Gen Comp Endocrinol. (2006) 148:260–72. 10.1016/j.ygcen.2006.03.012 16650423

[61] Pettis HM Rolland RM Hamilton PK Brault S Knowlton AR Kraus SD Visual health assessment of North Atlantic right whales *(Eubalaena glacialis)* using photographs. Can J Zool. (2004) 82:8–19. 10.1139/z03-207

[62] Perry J A Review of the Scientific Literature Concerning the Welfare of Wild Animals in the UK. Masters thesis, York University (2017).

[63] Wade PR Reeves RR Mesnick SL Social and behavioural factors in Cetacean responses to overexploitation: are odontocetes less “Resilient” than mysticetes? J. Mar. Biol. (2012) 2012:567276 10.1155/2012/567276

[64] CMS CMS Resolution 11.23: Conservation Implications of Cetacean Culture. (2015). Available online at: http://www.ascobans.org/sites/default/files/document/AC22_Inf_4.7.b_CMSres.11.23_CetaceanCulture.pdf (Accessed May 11, 2018).

[65] Wade PR Reilly SB Gerrodette T Assessment of the Population Dynamics of the Northeastern Offshore Spotted and the Eastern Spinner Dolphin Populations Through 2002. NOAA Report LJ-02-13 (2002). p. 53.

[66] Gerrodette T Forcada J Non-recovery of two spotted and spinner dolphin populations in the eastern tropical Pacific Ocean. Mar Ecol Prog Ser. (2005) 291:1–21. 10.3354/meps291001

[67] Cramer KL Perryman WL Gerrodette T Declines in reproductive output in two dolphin populations depleted by the yellowfin tuna purse-seine fishery. Mar Ecol Prog Ser. (2008) 369:273–85. 10.3354/meps07606

[68] Noren SR Edwards EF Physiological and behavioural development in Delphinid calves: implications for calf separation and mortality due to tuna purse-seine sets. Mar Mamm Sci. (2007) 23:15–29. 10.1111/j.1748-7692.2006.00083.x

[69] Noren SR Altered swimming gait and performance of dolphin mothers: implications for interactions with tuna purse-seine fisheries. Mar Ecol Prog Ser. (2013) 482:255–63. 10.3354/meps10286

[70] Gilman EL Bycatch governance and best practice mitigation technology. Mar Pol. (2011) 35:590–609. 10.1016/j.marpol.2011.01.021

[71] Mann J Smolker RA Smuts BB Responses to calf entanglement in free-ranging bottlenose dolphins. Mar Mamm Sci. (1995) 11:100–6. 10.1111/j.1748-7692.1995.tb00280.x

[72] Miketa ML Krzyszczyk E Mann J Behavioral responses to fishing line entanglement of a juvenile bottlenose dolphin in Shark Bay, Australia. Matters (2017) 1–6. 10.19185/matters.201711000011

[73] Whitehead H. Conserving and managing animals that learn socially and share cultures. Learn Behav. (2010) 38:329–36. 10.3758/LB.38.3.329 20628170

[74] Frère CH Krützen M Mann J Connor RC Bejder L Sherwin WB. Social and genetic interactions drive fitness variation in a free-living dolphin population. Proc Natl Acad Sci USA. (2010) 107:19949–54. 10.1073/pnas.1007997107 21041638 PMC2993384

[75] Möller L Harcourt RG Shared reproductive state enhances female associations in dolphins. Res Lett Ecol. (2008) 2008:498390 10.1155/2008/498390

[76] Bernaldo de Quirós Y Seewald JS Sylva SP Greer B Niemeyer M Bogomolni AL. Compositional discrimination of decompression and decomposition gas bubbles in bycaught seals and dolphins. PLoS ONE (2013) 8:e83994. 10.1371/journal.pone.0083994 24367623 PMC3868626

[77] Read FL Evans PGH Dolman SJ Cetacean Bycatch Monitoring and Mitigation under EC Regulation 812/2004 in the Northeast Atlantic, North Sea and Baltic Sea from 2006 to 2014. A WDC Report. (2017). 76 p.

[78] European Commission Communication from the Commission to the European Parliament and the Council-Cetacean Incidental Catches in Fisheries: Report on the Implementation of Certain Provisions of Council Regulation (EC) No 812/2004 and on a Scientific Assessment of the Effects of Using in Particular Gillnets, Trammel Nets and Entangling Nets on Cetaceans in the Baltic Sea as Requested Through Council Regulation (EC) No 2187/2005. (2009). p. 9 Available online at: http://eur-lex.europa.eu/procedure/EN/198455

[79] European Commission Communication from the Commission to the European Parliament the Council on the Implementation of Certain Provisions of Council Regulation (EC) (No812/2004) Laying Down Measures Concerning Incidental Catches of Cetaceans in Fisheries Amending Regulation (EC) (No88/98). (2011). Available online at: http://eur-lex.europa.eu/LexUriServ/LexUriServ.do?uriCOM:2011:0578:FIN:EN:PDF

[80] ICES Request from EU Concerning Monitoring of Bycatch of Cetaceans and Other Protected Species. (2013). Available online at: http://www.ices.dk/sites/pub/Publication%20Reports/Advice/2013/Special%20requests/EU_bycatch%20of%20cetaceans%20and%20other%20protected%20species.pdf

[81] ICES Bycatch of Small Cetaceans and Other Marine Animals – Review of National Reports Under Council Regulation (EC) No. 812/2004 and Other Published Documents. (2014). Available online at: http://www.ices.dk/sites/pub/Publication%20Reports/Advice/2014/2014/Bycatch_of_small_cetaceans_and_other_marine_animals.pdf

[82] ICES Report of the Workshop to Evaluate Aspects of EC Regulation 812/2004 (WKREV812). (2011). p. 67 Available online at: http://www.ascobans.org/sites/default/files/document/ICES_WKREV812_final-updated_2011.pdf

[83] ICES Bycatch of Small Cetaceans and Other Marine Animals – Review of National Reports Under Council Regulation (EC) No. 812/2004 and Other Information. (2016). Available online at: http://www.ices.dk/sites/pub/Publication%20Reports/Advice/2016/2016/Protected_species_bycatch.pdf

[84] European Commission Technical Conservation Measures Proposal. (2016). Available online at: https://ec.europa.eu/transparency/regdoc/rep/1/2016/EN/1-2016-134-EN-F1-1.PDF

[85] DG Environment Background Document on Relevant EU Policy Matters. Information paper 8a submitted to ASCOBANS 24^th^ Advisory Committee. (2018).

[86] European Parliament Conservation of Fishery Resources and the Protection of Marine Ecosystems Through Technical Measures. Amendments 230-582. Draft Report. Committee on Fisheries. 2016/0074(COD). (2017). p. 176.

[87] ASCOBANS Conservation Plan for the Harbour Porpoise Population in the Western Baltic, the Belt Sea and the Kattegat. (2012). Available online at: http://www.ascobans.org/sites/default/files/document/HarbourPorpoise_ConservationPlan_WesternBaltic_MOP7_2012.pdf (Accessed March 12, 2018).

[88] ASCOBANS Conservation Plan for Harbour Porpoises (Phocoena phocoena L.) in the North Sea. (2009). Available online at: http://www.ascobans.org/sites/default/files/document/ASCOBANS_NorthSeaPlan_MOP6.pdf (Accessed March 12, 2018).

[89] ASCOBANS Resolution No. 10: Small Cetacean Stranding Response. 8th Meeting of the Parties to ASCOBANS, Helsinki, Finland (2016b). Available online at: http://www.ascobans.org/sites/default/files/document/MOP8_2016-10_StrandingResponse.pdf (Accessed March 14, 2018).

[90] Dawson SM Northridge S Waples D Read AJ To ping or not to ping: the use of active acoustic devices in mitigating interactions between small cetaceans and gillnet fisheries. End. Sp. Res. (2013) 19:201–21. 10.3354/esr00464

[91] Carretta JV Barlow J Enriquez L Acoustic pingers eliminate beaked whale bycatch in a gill net fishery. Mar. Mam. Sci. (2008) 24:956–61. 10.1111/j.1748-7692.2008.00218.x

[92] Lepper PA Gordon J Booth C Theobald P Robinson SP Northridge S Establishing the Sensitivity of Cetaceans and Seals to Acoustic Deterrent Devices in Scotland. Scottish Natural Heritage Commissioned Report No. 517. (2014).

[93] Jaiteh VF Allen SJ Meeuwig JJ Loneragan NR Combining in-trawl video with observer coverage improves understanding of protected and vulnerable species by-catch in trawl fisheries. Mar. Fresh. Res. (2014) 65:830–37. 10.1071/MF13130

[94] van Beest FM Kindt-Larsen L Bastardie F Bartolino V Nabe-Nielsen J Predicting the population-level impact of mitigating harbor porpoise bycatch with pingers and time-area fishing closures. Ecosphere (2017) 8:e01785 10.1002/ecs2.1785

[95] Fernández-Contreras MM Cardona L Lockyer CH Aguilar A Incidental bycatch of short-beaked common dolphins (*Delphinus delphis*) by pairtrawlers off northwestern Spain. – ICES J. Mar. Sci. (2010) 67:1732–8. 10.1093/icesjms/fsq077

[96] Mannocci L Dabin W Augeraud-Véron E Dupuy J-F Barbraud C Ridoux V. Assessing the impact of bycatch on dolphin populations: the case of the common dolphin in the Eastern North Atlantic. PLoS ONE (2012) 7:e32615. 10.1371/journal.pone.0032615 22393423 PMC3290591

[97] Deaville R Annual report for the period 1^st^ January – 31^st^ December. UK Cetacean Strandings Investigation programme. 2015. (2015). Available online at: http://randd.defra.gov.uk/Document.aspx?Document=14001_FINALUKCSIPAnnualReport2015.pdf

[98] Australian Fisheries Management Authority Dolphin Strategy. Minimising gillnet bycatch. (2014). Available online at: https://www.afma.gov.au/sustainability-environment/protected-species-management-strategies (Accessed May 1/5/2018).

[99] Northridge S Kingston A Mackay A Lonergan M Bycatch of Vulnerable Species: Understanding the Process and Mitigating the Impacts. Final Report to Defra Marine and Fisheries Science Unit, Project no MF1003, University of St Andrews Defra, London, (2011). 99 p.

[100] Northridge S Cargill A Coram A Mandleberg L Calderan S Reid R Entanglement of Minke Whales in Scottish Waters; an Investigation into Occurrence, Causes and Mitigation. SMRU report to Scottish Government. (2010).

[101] Mathewson A Non-Lethal Entanglement of Minke Whales (Balaenoptera acutorostrata) in Fishing Gear in the Hebrides. Bachelors thesis submitted to University of St Andrews, Fife (2012).

[102] Knowlton AR Robbins J Landry S McKenna HA Kraus SD Werner TB. Effects of fishing rope strength on the severity of large whale entanglements. Conserv. Biol. (2015) 30:318–28. 10.1111/cobi.12590 26183819

[103] Katona SK Rough V Richardson DT A Field Guide to Whales, Porpoises and Seals from Cape Cod to Newfoundland. Washington, DC: Smithsonian Institution Press (1993).

[104] Leaper R Papastavrou V Sadler L Consideration of Factors Affecting Time to Death for Whales Following Entanglement in Fishing Gear. Paper IWC/58/WKMandAWI 14 presented to International Whaling Commission. (2006). 6 p.

[105] Camps FE Cameron JM Practical Forensic Medicine. 2nd ed. Vol. 316 London: Hutchinson Medical Publications (1971).

[106] DeMaster D Miller D Henderson JR Coe JM Conflicts between marine mammals and fisheries off the coast of California. In: Beddington J, Bever-ton RJH, Lavigne DM, editors. Marine Mammals and Fisheries. London: George Allen and Unwin (1985). p. 111–7.

[107] Kuiken T Simpson VR Allchin CR Bennett PM Codd GA Harris EA. Mass mortality of common dolphins (*Delphinus delphis*) in south west England due to incidental capture in fishing gear. Vet Record. (1994) 134:81–9. 10.1136/vr.134.4.81 8178416

[108] Jepson PD Baker JR Kuiken T Simpson VR Kennedy S Bennett PM. Pulmonary pathology of harbour porpoises (*Phocoena phocoena*) stranded in England and wales between 1990 and 1996. Vet Record. (2000) 146:721–8. 10.1136/vr.146.25.721 10901214

[109] Duignan PJ Gibbs NJ Jones GW Autopsy of Cetaceans Incidentally Caught in Fishing Operations 1997/98, 1999/2000, and 2000/01 Doc Science Internal Series. New Zealand Department of Conservation (2004). p. 119.

[110] Garner R Lyons D Roberts A How to Protect Animal Welfare: Deliberation, Democracy and Representation. Centre for Animals and Social Justice. (2015).

[111] Lewison RL Crowder LB Read AJ Freeman SA Understanding impacts of fisheries bycatch on marine megafauna. TREE (2004) 19:598–604. 10.1016/j.tree.2004.09.004

[112] Bisack KD Das C Understanding non-compliance with protected species regulations in the Northeast USA gillnet fishery. Front. Mar. Sci. (2015) 2:91 10.3389/fmars.2015.00091

[113] Teh LSL Teh LCL Hines E Junchompoo C Lewison RL Contextualising the coupled socio-ecological conditions of marine megafauna bycatch. Ocean Coast Manage. (2015) 116:449–65. 10.1016/j.ocecoaman.2015.08.019

[114] Read AJ The looming crisis: interactions between marine mammals and fisheries. J Mar Mam. (2008) 89:541–8. 10.1644/07-MAMM-S-315R1.1

[115] Marine Stewardship Council MSC sets out agenda to strengthen assurance model revisions to certification requirements. Press release. (2018). Available online at: https://www.msc.org/media-centre/press-releases/msc-reveals-scope-of-next-fisheries-standard-review (Accessed May 11, 2018).

